# Impact of Pediatric Obesity on Diurnal Blood Pressure Assessment and Cardiovascular Risk Markers

**DOI:** 10.3389/fped.2021.596142

**Published:** 2021-03-04

**Authors:** Margaret O. Murphy, Hong Huang, John A. Bauer, Aric Schadler, Majd Makhoul, Jody L. Clasey, Aftab S. Chishti, Stefan G. Kiessling

**Affiliations:** ^1^Division of Pediatric Nephrology, Department of Pediatrics, University of Kentucky, Lexington, KY, United States; ^2^Department of Pediatrics, University of Kentucky, Lexington, KY, United States; ^3^Division of Pediatric Cardiology, Department of Pediatrics, University of Kentucky, Lexington, KY, United States; ^4^Department of Kinesiology and Health Promotion, University of Kentucky, Lexington, KY, United States

**Keywords:** blood pressure, pediatric, obesity, cardiovascular risk, nocturnal dipping, left ventricular hypertrophy

## Abstract

**Background:** The prevalence of hypertension is increasing particularly among obese children and adolescents. Obese children and adolescents with hypertension are likely to remain hypertensive as they reach adulthood and hypertension is linked to an increased risk for cardiovascular disease. Twenty-four-hour ambulatory blood pressure monitoring (ABPM) has become one of the most important tools in diagnosing hypertension in children and adolescents and circadian patterns of blood pressure may be important disease-risk predictors.

**Methods:** A retrospective chart review was conducted in patients aged 6–21 years who underwent 24-h ABPM at Kentucky Children's Hospital (KCH) from August 2012 through June 2017. Exclusion criteria included conditions that could affect blood pressure including chronic kidney disease and other renal abnormalities, congenital heart disease, cancer, and thyroid disease. Subjects were categorized by body mass index into normal (below 85th percentile), overweight (85th−95th percentile), stage I obesity (95th−119th percentile), stage II obesity (120th−139th) and stage III obesity (>140th). Non-dipping was defined as a nocturnal BP reduction of <10%.

**Results:** Two hundred and sixty-three patients (156 male patients) were included in the analysis, of whom 70 were normal weight, 33 overweight, 55 stage I obesity, 53 stage II, and 52 stage III obesity. Although there was no significant difference between normal weight and obese groups for prevalence of hypertension, there was a greater prevalence of SBP non-dipping in obese patients as BMI increased (*p* = 0.008). Furthermore, non-dippers had a significantly elevated LVMI as well as abnormal lab values for uric acid, blood lipid panel, creatinine, and TSH (*p* < 0.05).

**Conclusions:** These findings demonstrate that obese children and adolescents constitute a large proportion of hypertensive children and adolescents and the severity of pediatric obesity is associated with nocturnal BP non-dipping. Additionally, obesity in children is linked to several cardiovascular risk factors including left ventricular hypertrophy, dyslipidemia, and elevated uric acid levels. Further studies utilizing ABPM measures on risk stratification in this very high-risk population are warranted.

## Introduction

Childhood obesity and associated cardiometabolic disease including hypertension are increasing at an alarming rate worldwide. Currently 18.5% of children and adolescents (~13.7 million) are obese in the United States with rates of obesity increasing in all age groups in the pediatric population (1). A recent systematic review and meta-analysis examining the global prevalence of childhood hypertension reports that hypertension has increased from 1994 to 2018 with a pooled estimate of 4% and is associated with high body mass index (2). According to the National Health and Nutrition Examination Survey (NHANES) the prevalence of elevated blood pressure in children between the ages of 8–17 is reported to be 19% in boys and 13% in girls which is a 24% increase from 1988 to 1994 (3). This is an alarming report since pediatric blood pressure is a strong predictor of adult hypertension (4). Similarly, childhood obesity has been shown to project into adulthood (5, 6) leading to increased risk for cardiovascular disease such as heart failure, stroke, and myocardial infarction.

Pediatric hypertension has been associated with target-organ damage including left ventricular hypertrophy (LVH) (7), impaired cognition (8), and subclinical markers of cardiovascular disease such as increased carotid intima thickness (9). Risk factors for pediatric hypertension have not clearly been defined; however, children with obesity are at increased risk (10) as well as children with chronic kidney disease (11).

Ambulatory blood pressure monitoring (ABPM) provides a comprehensive assessment of blood pressure over a 24-h period and is recommended by the 2017 AAP CPG for the confirmation of the diagnosis of hypertension. Additionally, ABPM is recommended for the evaluation for masked hypertension, suspected white-coat hypertension, risk for hypertensive target organ damage, evaluation of hypertension in children with obstructive sleep apnea syndrome, secondary hypertension, chronic kidney disease or structural renal abnormalities, type 1 and type 2 diabetes, history of solid organ transplant, history of pre-maturity and evaluation of non-dipping status, as well as patients with history of aortic coarctation repair, in addition to assessment and monitoring of treatment effectiveness of antihypertensive medications (12). Strojny et al. assessed ABPM as a diagnostic tool in hypertension in children as well as prevalence of metabolic syndrome and found altered circadian blood pressure patterns in 50% of obese patients (13).

Traditional BMI categories of normal weight vs. overweight and obesity have been widely accepted as screening tools for cardiometabolic risk because of their simplicity, cost-effectiveness, and strong correlation with cardiac magnetic resonance imaging (CMR). Within the previous two decades, there has a been a significant increase in the prevalence of extreme obesity as defined by a BMI of at least 99th percentile on the CDC growth charts (14). Due to this increased adiposity, Flegal et al. have developed further classification schemes for extreme obesity by using modeling to derive an extended growth curve percentile for children. The additional sub-categories include class I (BMI is 95th to 120% of the 95th percentile), class II (BMI 120–140% of the 95th percentile), and class III [BMI>140% of the 95th percentile (15)]. In 2013, The American Heart Association and the Endocrine Society Guidelines on Childhood Obesity endorsed the definition of extreme obesity in children 2 years of age and older as a BMI > 120% of the 95th percentile or an absolute BMI > 35 kg/m^2^ (16). Cardiovascular risk factors associated with pediatric obesity include dyslipidemia, hypertension, insulin resistance, and the metabolic syndrome, as well as other non-traditional risk factors including increased levels of homocysteine, uric acid, and C-reactive protein (14).

Left ventricular hypertrophy (LVH) is readily assessed by echocardiography and is a form of target organ damage (17). The AAP CPG defines LVH as LV mass >51 g/m^2.7^ for children older than 8 years of age and recommends echocardiograms be obtained when the initiation of antihypertensive medication is considered to assess target organ damage (18). ABPM has been shown to correlate with target organ damage such as left ventricular hypertrophy in adults (19). LVH has been shown to be related to obesity independent of blood pressure changes (20).

The aim of this study was to evaluate relationships of elevated blood pressure and several established markers of cardiovascular and metabolic disease among patients stratified according to obesity and treated at the KCH Pediatric Nephrology Clinic. Furthermore, this study tests the hypothesis that severity of obesity correlates with increased cardiovascular risk including non-dipping and left ventricular hypertrophy.

## Materials and Methods

### Research and Design

This study was conducted as a retrospective chart review in children who were referred to the KCH Pediatric Nephrology Clinic with elevated blood pressure who underwent a first time 24-h ABPM between August 2012 through June 2017. This study evaluates the relationship between blood pressure and several established markers of cardiovascular and metabolic disease based on severity of obesity. This study was approved by the University of Kentucky Institutional Review Board.

### Selection Criteria and Outcomes Reported

Patients were selected *via* the electronic medical record to identify those patients who underwent a 24-h ABPM from KCH during the study timeframe. Exclusion criteria included conditions that could affect blood pressure including chronic kidney disease, congenital heart disease, cancer patients, patients with ADHD on stimulant medication, and thyroid disease. Data collected included demographic (date of birth, gender, ethnicity/race) and diagnostic evaluation (age at presentation, ABPM report, diagnostic testing including echocardiogram, and laboratory findings) from the electronic medical record.

All ABPMs were performed using Spacelabs (Issaquah, WA) 90217 monitor or an Oscar 2 ABPM Monitor by SunTech Medical which are validated by the Association for the Advancement of Medical Instrumentation for use in children. The monitor was placed by trained nursing staff at the time of clinic visit with an appropriate cuff size. Readings were taken every 30 min while awake and every 60 min while asleep. Patients were asked to record sleep and wake times during the 24-h period and were instructed to mail this information along with the device back to our office for interpretation of results. Patients were advised to follow routine daily activities but avoid vigorous exercise and to relax the arm during inflation and deflation of the cuff. The adequacy of the study was determined by the interpreting physician at the time of ABPM evaluation according to the Ambulatory Blood Pressure Monitoring in Children and Adolescents: Recommendations for Standard Assessment and included at least 65% successful readings (21). Patients were categorized according to the ABPM definition as normal, pre-hypertension, ambulatory hypertension, or severe hypertension (21). Non-dipping was defined as a nocturnal BP reduction of <10% from mean systolic and diastolic awake BP.

Patients' gender, age, height, and weight were measured at the clinic visit. BMI was calculated and plotted on CDC growth chart for gender and age. Data were available for BMI determination in all subjects. Subjects were categorized by body mass index into normal (below 85th percentile), overweight (85th−95th percentile), stage I obesity (95th−119th percentile), stage II obesity (120th−139th) and stage III obesity (>140th) based on the updated CDC growth charts.

Laboratory assessment including renal function panel, blood lipid panel (total cholesterol, HDL, LDL, triglycerides), hemoglobin A1c, uric acid, and renin-aldosterone-cortisol, and thyroid levels were obtained retrospectively. Cardiac imaging *via* echocardiogram to determine evidence of target organ damage was reported as well. Left ventricular hypertrophy (LVH) was defined as LV mass indexed to height in meters to the 2.7 power that is greater than the 95th percentile for age and sex (22).

### Statistical Analysis

Bivariate statistical analyses were performed *via* IBM SPSS Statistics version 25. (IBM Corp., Armonk, NY, USA) with significance set at an alpha level <0.05. Modeling was performed in SAS version 9.4 (SAS Institute Inc., Cary, NC, USA). Continuous variables including lab values such as blood lipid panel, hemoglobin A1c, uric acid, thyroid, renin-aldosterone-cortisol, and LVMI were analyzed using ANOVA utilizing a *post-hoc* pairwise comparison with a Bonferroni correction and independent samples *t*-test. Categorical and ordinal variables including gender, BMI stage, blood Pressure stage were analyzed using Pearson's chi-square while non-dipping prevalence were analyzed through Somer's D-test. Additionally, a generalized linear multivariate model was used to examine the interaction of BMI stage and gender on LVMI with significance set at an alpha level <0.05.

## Results

A total of 496 completed ABPMs were identified during the study period August 2012 through June 2017; 100 were excluded due to an underlying chronic condition as described in the methods section, an additional 46 of these were repeat ABPMs, and 87 were excluded due to inadequate sample collection (completed readings below 65%). Of those remaining, 263 ABPMs and associated patient data were included in the analysis, of whom 70 (27%) were normal weight, 33 (13%) were overweight, 55 (20%) were class I obesity, 53 (20%) were class II obesity, and 52 (20%) were class III obesity. The mean age was 13.9 ± 0.17 with 98% patients (*n* = 259) 18 years or younger, 40% are female and there were no significant differences among gender and age in each obesity class. Demographics including age, gender, race, and mean BMI are found in Table 1.

**Table 1:** Patient characteristics.

	**Normal**	**Overweight**	**OB Class I**	**OB Class II**	**OB Class III**
*N* %	70 (27)	33 (13)	55 (20)	53 (20)	52 (20)
Age (yr), Mean ± SD	14.1 ± 2.9	14.5 ± 2.5	14.1 ± 2.90	13.5 ± 2.55	13.3 ± 2.8
**Gender**					
Male, *n* (%)	35 (50)	19 (58)	28 (51)	39 (74)	37 (72)
Female, *n* (%)	35 (50)	14 (42)	27 (49)	14 (27)	15 (29)
Race					
Caucasian, *n* (%)	58 (83)	27 (82)	53 (97)	45 (83)	40 (77)
African American, *n* (%)	10 (15)	6 (18)	2 (3)	5 (10)	11 (22)
Other, *n* (%)	2 (2)	0 (0)	0 (0)	0 (0)	1
Unreported, *n* (%)	0 (0)	0 (0)	0 (0)	1	3 (6.5)
BMI (kg/m^2^), Mean ± SD	20.2 ± 3.1	25.3 ± 2.4	29.4 ± 3.11	33.6 ± 3.64	39.8 ± 5.6

**Figure 1:**
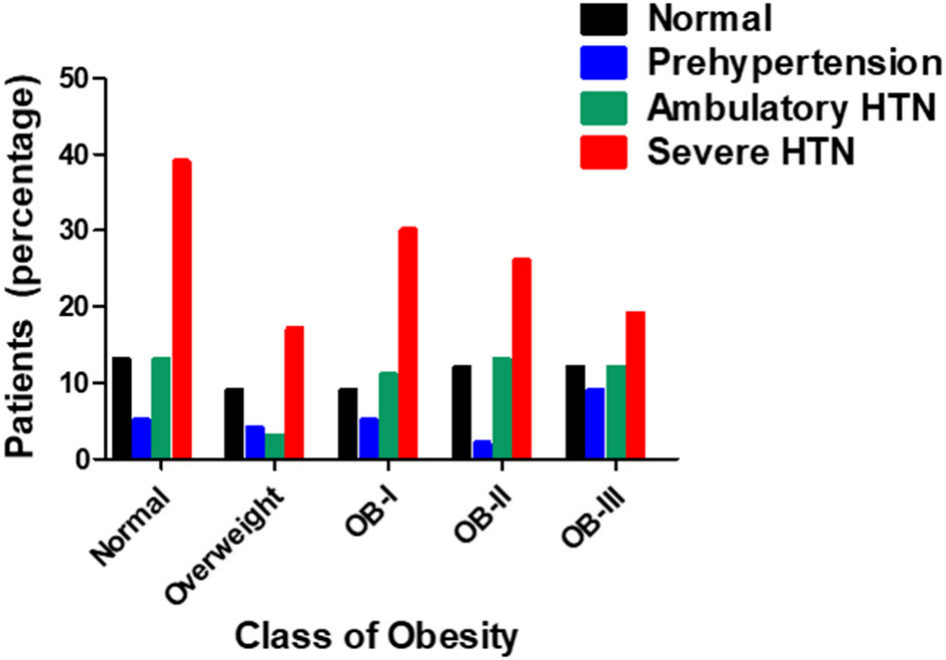
Prevalence of hypertension in relation to stage of obesity (*n* = 263) as tested by Pearson's chi square-test.

**Figure 2:**
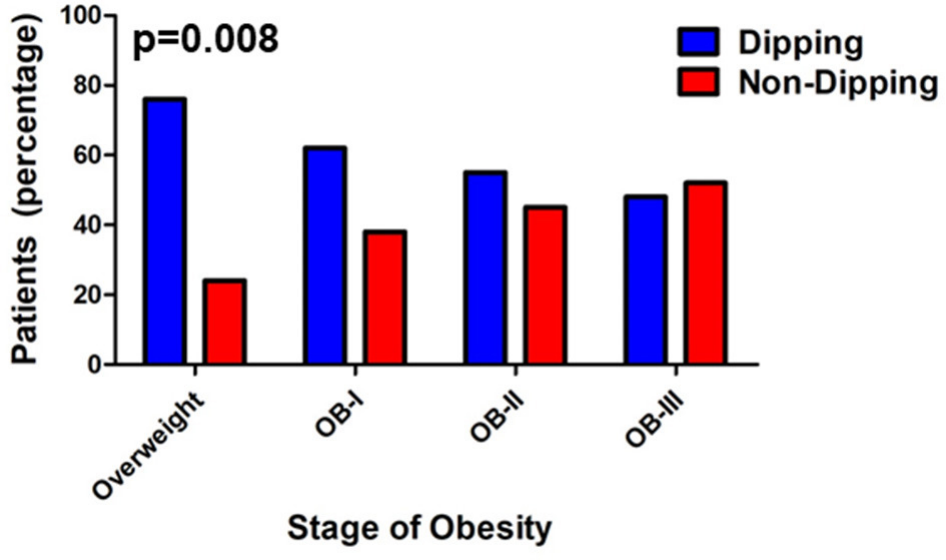
Prevalence of non-dipping increases with severity of obesity. Values are presented as percentage with Somer's D-test (*p* = 0.008, Somer's D-test).

ABPM findings including 24-h systolic blood pressure and diastolic blood pressure, as well as mean day and night SBP and DBP according to obesity class are reported in Table 2 with the number of successful readings during sleep in each category is as follows: normal weight:9.64 ± 0.469, overweight: 8.25 ± 0.651, stage I obesity: 8.95 ±0.492, stage II: 8.44 ± 0.371, stage III: 8.32 ± 0.582 (*p* = 0.161). The different stages of hypertension as defined by ABPM which takes into account the mean ambulatory systolic blood pressure and load were not significantly different among obesity classes (*p* = 0.161, Figure 1). Prevalence of non-dipping (defined as nocturnal BP reduction of <10%) significantly increased as severity of obesity increased for systolic blood pressure (*n* = 8 in overweight; *n* = 21 in stage I obesity, *n* = 24 in stage II obesity, *n* = 27 in stage III obesity, *p* = 0.008; Figure 2); however, no differences were noted in nocturnal diastolic blood pressure dip (*p* = 0.15, Table 2).

**Table 2:** ABPM results.

	**Normal *N* = 70**	**Overweight*N* = 33**	**OB Class I *N* = 55**	**OB Class II*N* = 53**	**OB Class III *N* = 52**	***P*-value**
24-h SBP	131.1 ± 1.33	132.13 ± 2.46	134.30 ± 2.04	134.37 ± 1.72	131.55 ±1.51	0.503
24-h DBP	75.03 ± 1.22	72.72 ±1.12	72.76 ±1.17	72.28 ± 0.99	70.70 ± 1.17	0.0951
Day SBP	134.64 ± 1.35	134.78 ± 2.05	137.02 ±1.73	137.02 ±1.73	135.42 ±1.84	0.703
Day DBP	78.44 ±1.14	75.88 ±1.16	76.31 ±1.24	75.28 ±1.06	73.0 ±1.kh	**0.015**
Night SBP	120.14 ±1.66	119.76 ± 2.45	119.89 ± 1.62	123.45 ± 2.16	121.96 ± 1.54	0.565
Night DBP	64.78 ± 1.31	61.15 ± 1.27	61.12 ± 0.96	62.19 ± 1.20	60.71 ± 0.88	0.056
Nocturnal SBP non-dipping, *n* (%)	31 (44.3%)	8 (24.2%)	21 (38.2%)	24 (45.3%)	27 (51.9%)	**0.008**
Nocturnal DBP non-dipping, *n* (%)	15 (21.4%)	4 (12.1%)	6 (10.9%)	9 (17%)	7 (13.7%)	0.1539
Number of sleep readings	9.64 ± 0.469	8.25 ± 0.615	8.95 ±0.492	8.44 ± 0.371	8.32 ± 0.582	0.161

*Bolded value indicates a p < 0.05*.

**Figure 3:**
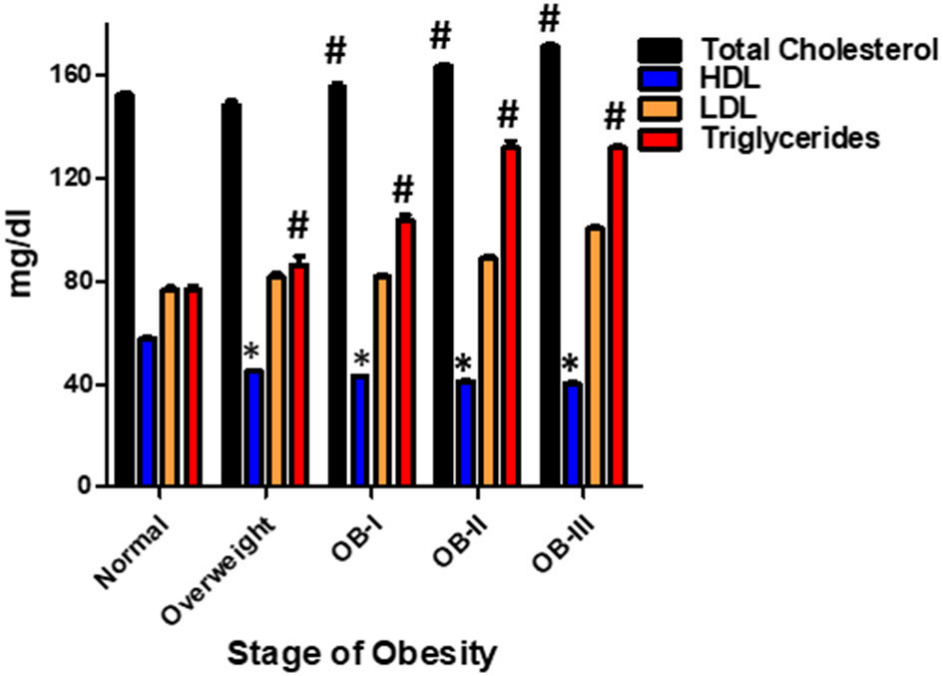
Lipid profile based on stage of obesity. Values are presented as mean ± SEM with One-Way ANOVA-test as appropriate (#*p* < 0.001 for total cholesterol, triglycerides; *p < 0.05 for HDL compared to normal weight group).

**Figure 4:**
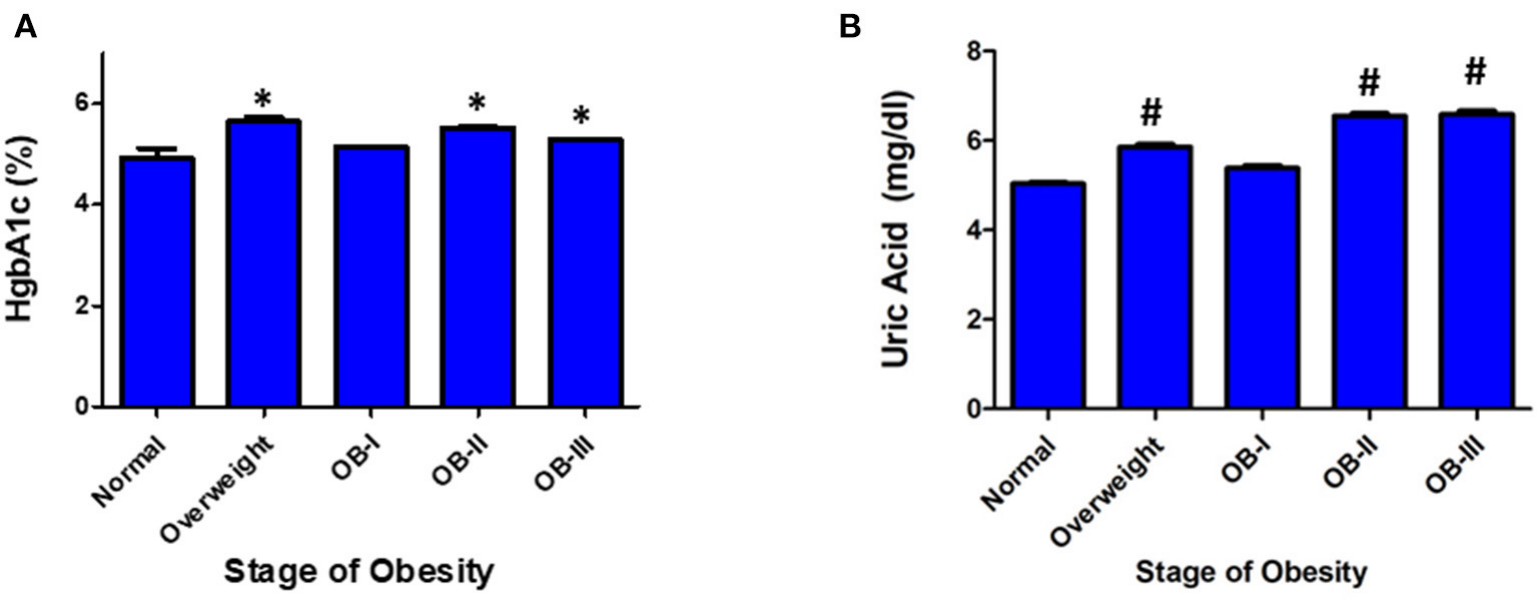
Serum markers of glucose metabolism and cardiovascular risk. **(A)** HgbA1C and **(B)** Uric Acid Values are presented as mean ± SEM with One-Way ANOVA-test as appropriate (#*p* < 0.001 for uric acid; *p < 0.05 for HgbA1C compared to normal weight group).

**Figure 5:**
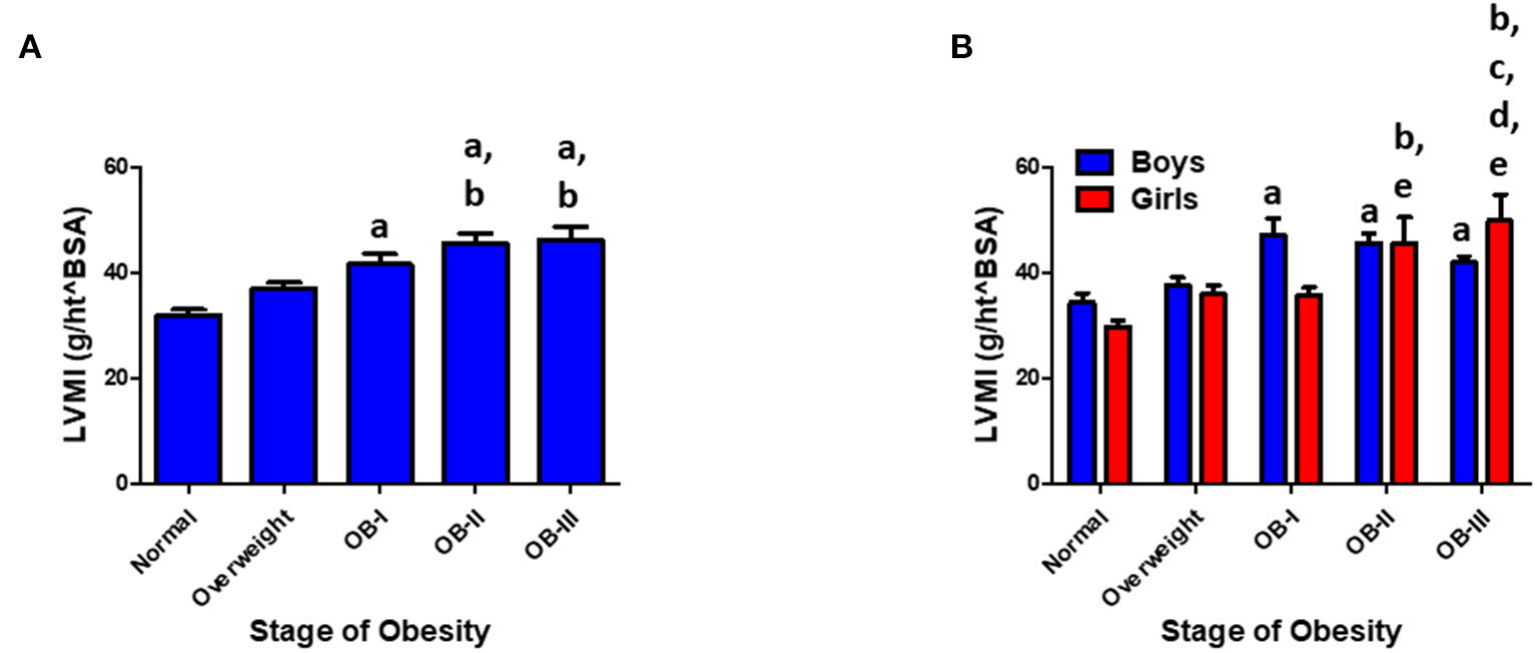
**(A)** LVMI increases with stage of obesity. Values are presented as mean ± SEM with One-Way ANOVA-test with Bonferroni *post-hoc* analysis ^a^*p* < 0.001 compared to normal weight; ^b^*p* < 0.001 compared to overweight group. **(B)** Gender and LVMI. ^a^*p* < 0.001 compared to normal weight for boys. ^b^*p* < 0.001 compared to normal weight for girls; ^c^*p* < 0.05 compared to overweight for girls; ^d^*p* < 0.05 compared to stage I obesity for girls; ^e^*p* < 0.05 compared to stage II obesity for girls. Interaction between gender and obesity rank: *p* < 0.05.

Of the patients who underwent ABPM measurement and included in analysis, 234 patients had a renal function panel measured and 178 had lipid panel measured. Total cholesterol was significantly elevated as severity of obesity increased (*p* < 0.001) in addition to significant increases in serum triglyceride (*p* < 0.001, Figure 3). There was an inverse relationship between HDL and severity of obesity (*p* < 0.001). Levels of uric acid were found to be significantly elevated with stage II and III obesity (*p* < 0.001). HgbA1c was measured in 109 patients and found to be elevated in overweight and obese groups compared to normal weight groups (*p* < 0.05, Figure 4). No statistical differences were found in measurements of renal function, vitamin D levels, or the hormones renin, aldosterone, and cortisol across classes of obesity (Supplementary Figure 1). TSH was significantly elevated in class II and III obesity suggesting a hypothyroid phenotype (*p* < 0.05). Of those patients who underwent ABPM measurement and included in analysis, 197 patients underwent echocardiogram (117 were male) with 6% (*n* = 11) diagnosed with LVH. Left ventricular mass index assessed *via* echocardiogram revealed a significant elevation with severity of obesity (normal weight: 33.95 ± 0.41; overweight: 43.4 ± 1.07, stage I: 50.4 ± 1.77; stage II: 52.64 ± 1.79; stage III: 57.9 ± 1.8, *p* < 0.001, Figure 5A). Further examination reveals that the effect of LVMI is correlated with obesity stage (Figure 5A) but not with blood pressure level (one-way ANOVA, *p* = 0.709) or non-dipping (Chi square-test, *p* = 0.721). When we further analyzed the difference in LVMI, we found an interaction between LVMI and gender. Although normal weight girls have a lower LVMI than normal weight boys, LVMI has higher values for girls in stage III obesity than boys (49.89 ± 4.92 vs. 45.09 ± 2.92, Figure 5B, *p* = 0.030).

## Discussion

This study examined several hemodynamic and biochemical markers of cardiovascular risk with respect to obesity class and demonstrated that the prevalence of non-dipping during 24h BP monitoring was more prevalent with increasing severity of obesity. Furthermore, several cardiovascular risk markers including total cholesterol, triglycerides, uric acid levels, and LVMI were significantly associated with severity of obesity. We found a potentially important trend of increased prevalence of non-dipping and greater left ventricular mass in the most severe obesity classes.

Nocturnal dip is defined as a nocturnal blood pressure reduction of <10% from mean systolic and diastolic awake BP during the nighttime. The relationships between non-dipping status and target organ damage and cardiovascular morbidity is well-established in adults. Non-dipping in the pediatric population has been less studied but reveals associations with target organ damage such as LVH in diabetic populations (23) and a lower GFR in children with chronic kidney disease (24). Framme et al. examined dipping status and obesity in children and found a significant impact only in females (25) whereas our findings found a relationship in both genders as well as reported by others (26). Our findings reveal of prevalence of 45% non-dipping among obese patients compared to 34% by Macumber's study and 40% by Westerstahl's study (27). Furthermore, we report a positive association between severity of obesity and non-dipping status. The relationship between nocturnal dipping and obstructive sleep apnea in children remains mixed (28, 29); however; it is likely that our study included patients with undiagnosed sleep apnea.

Similar to our findings, the ERICA study from Brazil reports an increase in prevalence of several cardiovascular risk factors including total cholesterol, LDL cholesterol, low HDL levels, and HgbA1c in relation to severity of obesity in addition to blood pressure (30). Skinner at al. demonstrated that children aged 3–19 years of age with class III obesity had a >2-fold increased risk of hyperglycemia and hypertension (31). Data from NHANES of 20,905 youth aged 6–19 years revealed that odds for elevated blood pressure, hypercholesterolemia, and fasting hyperglycemia progressively doubled with each stage of obesity (32). Another study of severely obese adolescents enrolled at a National Obesity Center in Sweden examined the relationship between insulin-glucose metabolism, nocturnal blood pressure dipping and cardiac left ventricular mass and found that non-dipping was common and negatively associated with measures of insulin metabolism including Homeostatic Model Assessment of Insulin Resistance (HOMA) index and fasting insulin (27). They did not find associations between dipping status and HgA1c or LVMI. Since the HOMA index primarily reflects insulin levels, this suggests that high insulin levels could be important in the pathogenesis of non-dipping and should be monitored closely. Others have found that impaired glucose tolerance triples the risk of non-dipping among normotensive adults without diabetes (33).

Published findings in adult populations have demonstrated an association between serum uric acid and increased CVD mortality independent of traditional risk factors (34). A prospective 1 year study of 53 children and adolescents with hypertension found relationships between elevated uric acid and cardiovascular risk factors including adiposity, low HDL levels, CRP, and left ventricular hypertrophy; however, there were was no association between uric acid and hypertension (35). A more recent study with 333 obese youth aged 5–18 years examined the relationship between uric acid, ABPM, and other cardiometabolic risk factors and found a positive association between uric acid levels and blood pressure, insulin, and triglyceride levels (36). Our findings demonstrate a positive relationship between uric acid and severity of obesity.

The level and duration of BP elevation that result in target organ damage in children and adolescents remain poorly defined; however, LVH has been noted in this population with even mild blood pressure elevation and is probably the most well-studied marker of target organ damage because of the wide availability of echocardiography. In hypertensive children, the prevalence of LVMI ranges from 4.8 to 50% and can be even higher in children on dialysis (37–39). Daniels et al. reported that 47% of pediatric patients with hypertension also had a LVMI greater than the 95th percentile for LVMI in normal children and 8% had a LVMI >51 g/m^2.7^ (17). In a European study, it was found that pediatric patients with a new diagnosis of hypertension also had a 41% prevalence of LVH with 13.2% exhibiting a LVMI greater than the adult cutoff point (Litwin et al.). LVMI has been linked with ambulatory blood pressure parameters including nighttime SBP, 24 h blood pressure, and pulse pressure (40, 41). Similar to our findings, Ramaswamy et al. report a relationship between obesity and elevated LVMI independent of blood pressure in children who underwent 24 h blood pressure monitoring (42). In a different study that included children with secondary forms of hypertension, relationships were found between LVMI and elevated BMI and boys had a significantly higher LVMI than girls (43). However, this study did not examine whether an interaction existed between obesity stage and gender and it included more severe stages of hypertension due to secondary causes including chronic kidney disease and renovascular disease. Interestingly, our data is similar to these findings in normal weight individuals; however, we report significant elevations in LVMI in girls with more severe obesity. Results from the CKiD (chronic kidney disease cohort in children) have reported a four-fold odds of LVH among girls compared to boys (44). Additionally, studies in young adults have reported that women with hypertension were more likely to develop LVH than men when adjusting for ambulatory BP and other CVD risk factors (45). We note that our findings of differences between girls and boys in relation to LVMI and obesity is an interesting finding and warrants further studies with larger sample sizes.

Several studies have demonstrated that childhood cardiovascular risk factors carry forward into adulthood. A meta-analysis that examined the association between childhood obesity and cardiovascular risk in adulthood found a significant and positive association in regard to adult systolic blood pressure, diastolic blood pressure, triglycerides, and negatively associated with adult HDL (46). Furthermore, the Princeton Follow-up study has shown that pediatric hypertriglyceridemia can predict cardiovascular disease events in the fourth to fifth decade of life (47). A recent study utilizing CVD prediction algorithms from clinical data including serum lipids, systolic blood pressure, diabetes status, and BMI demonstrated that adolescents with severe obesity have a significant risk of having a cardiovascular event before the age of 50; however, after undergoing bariatric surgery, the predicted risk of CVD events was significantly reduced (48). Extrapolating these results into our own findings suggests that with severity of obesity, the likelihood of a cardiovascular event in adulthood is increased.

Our study has several limitations including that it is a single center retrospective experience and a cross-sectional design. Since this study is cross-sectional and included a single time point per study participant as a retrospective study we cannot infer causality between different variables examined; rather these relationships can guide additional prospective studies. In addition, we did not collect parameters related to sodium intake or physical activity patterns, which could affect blood pressure findings. All of our patients were referred to our clinic for hypertension evaluation, which may reduce the generalizability to the pediatric population. An additional factor is the tolerability to wearing the 24 h blood pressure monitor as it has been reported that obese adolescents have less successful readings and are thus more intolerant to ABPM (49). This could have affected our results since 87 subjects (33% of the overall sample) who underwent the ABPM-test were excluded due to inadequate readings and this could have influenced the findings on nocturnal dip and cardiovascular risk factors. An additional limitation of this study is that we did not account for sleep quality through sleep studies within these patients. Despite these limitations, we feel that this study adds significant findings to the literature regarding pediatric hypertension and cardiovascular risk based on severity of obesity as a way to stratify risk.

## Conclusions

Pediatric obesity is a major health concern and obese children are at a high risk of early cardiovascular disease. Recent strategies to stratify severity of obesity may provide opportunity to identify patients at highest risk. Our investigations of several hemodynamic and biochemical markers of cardiovascular risk with respect to Obesity Class provided the following insights: the frequency of systolic or diastolic hypertension was not different among obesity severity, but the prevalence of non-dipping during 24h BP monitoring was more prevalent with severity of obesity. Several well-recognized markers of cardiovascular risk are related to severity of obesity. Blood lipid panels demonstrated elevation in total cholesterol with a reduction in HDL, and elevation in triglycerides in relation to severity of obesity. LVMI was significantly associated with severity of obesity and this was found to be more prominent in girls than boys. Our data suggests that there may be a sex-specific target organ response and that obesity class may help to identify the most at-risk children at the earliest times. We found a potentially important trend of increased prevalence of non-dipping and greater LV mass in the most severe stages of obesity. Our studies support the use of ABPM in the evaluation of pediatric hypertension as well as the need to examine additional cardiovascular risk factors at the time of evaluation. Further studies to develop easily implemented and cost-effective risk stratification methods are clearly warranted as are studies to define relationships among blood pressure variables and cardiac structure and function in high-risk obese children.

## Data Availability Statement

The raw data supporting the conclusions of this article will be made available by the authors, without undue reservation.

## Ethics Statement

The studies involving human participants were reviewed and approved by University of Kentucky Institutional Review Board. Written informed consent from the participants' legal guardian/next of kin was not required to participate in this study in accordance with the national legislation and the institutional requirements.

## Author Contributions

MM designed the study, collected data, created figures after analysis, drafted the initial manuscript, and reviewed and revised the manuscript. AC and SK also assisted in the design of the study and collection of the data. HH, MM, JB, and JC oversaw the design and analysis of the study as well as supervising data analyses, review, and revision of the manuscript. AS provided statistical analysis support and reviewed the manuscript. SK designed the study, supervised the data analyses, and critically reviewed and revised the manuscript. All authors contributed to the manuscript review and revision and approved the submitted version.

## Conflict of Interest

The authors declare that the research was conducted in the absence of any commercial or financial relationships that could be construed as a potential conflict of interest.

## Supplementary Material

Supplementary Figure 1 Laboratory tests values are presented as mean ± SEM with One-Way ANOVA-test.
