# Supplementary figures and images for: Impact of Pediatric Obesity on Diurnal Blood Pressure Assessment and Cardiovascular Risk Markers
Source: Front Pediatr. 2021 Mar 4;9:596142. doi: 10.3389/fped.2021.596142 (PMC7969716; doi:10.3389/fped.2021.596142)

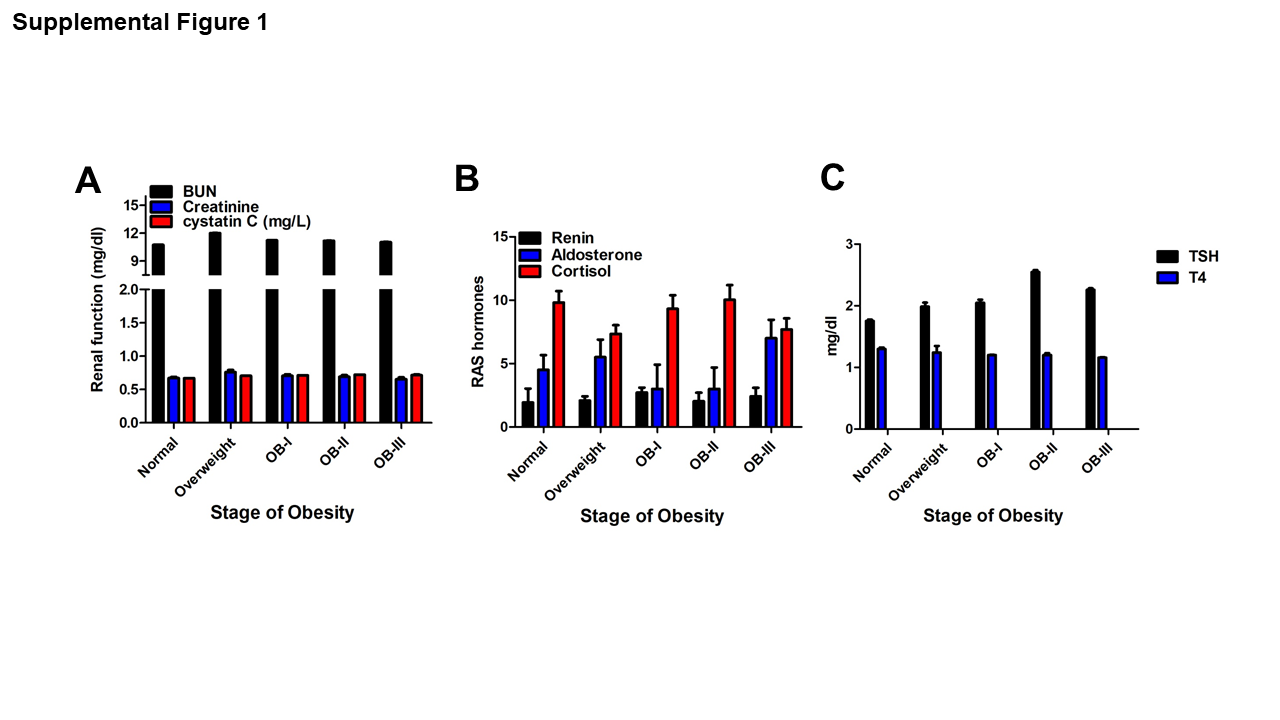

Supplement: Supplementary Figure 1 — Laboratory tests values are presented as mean ± SEM with One-Way ANOVA-test. [file Image_1.TIF]
